# The psychological impact on mothers who have experienced domestic violence when navigating the family court system: a scoping review

**DOI:** 10.1080/13218719.2023.2214927

**Published:** 2023-07-04

**Authors:** Sage Wilde, Nicola Sheeran, Heather Douglas

**Affiliations:** a School of Applied Psychology, Griffith University, Mount Gravatt, QLD, Australia; b Melbourne Law School, University of Melbourne, Melbourne, VIC, Australia

**Keywords:** coercive control, domestic violence, family court, family violence, intimate partner violence, legal, mother, psychological impact, systems abuse

## Abstract

The aim of this scoping review was to synthesise the literature to identify what the psychological impacts of family court processes were on mothers who had experienced DFV. Twenty-five articles met inclusion criteria with four themes capturing the findings: Perpetrators using the system as a mode of coercive control; Secondary victimisation as a result of interacting with the system; Required to relive their abuse; and, Long-term psychological consequences of having engaged with the system. Key findings were that perpetrators manipulated the system to perpetrate further abuse and continue/reassert their control. Secondary re-victimisation was common, with poor knowledge of DFV and limited understanding of coercive control tactics and how these were employed by perpetrators by legal professionals identified as contributing factors. This review suggests that mothers who engage with the family court system experience a range of short- and long-term psychological impacts and court processes facilitate ongoing abuse by the perpetrator.

Domestic and family violence (DFV) is a significant public health issue (World Health Organization, 2013) that disproportionately affects women (Australian Institute of Health and Welfare, AIHW, 2018). DFV is defined as threatening or violent behaviour that controls or coerces a family member, or causes fear (Australian Government, 2019). Violent and threatening behaviours can include physical violence, sexual abuse, emotional abuse, psychological abuse, economic control and any other behaviour that causes a person to live in fear (Department of Families, Fairness and Housing State, Government of Victoria, Australia, 2018). Coercive control has been recognised as a critical component of DFV and refers to the systematic behaviour pattern that establishes a person’s dominance through isolation, intimidation or threats of violence, often leading victims to be segregated from family, friends and support structures (Dichter et al., 2018).

In Australia, 23% of women have experienced emotional abuse from either their current partner or previous partners, while approximately 17% have experienced sexual or physical violence from a current or cohabitating partner (AIHW, 2018). While DFV is not limited to age, demographic or socioeconomic groups, women between 25 and 44 years are significantly at risk, with DFV being the most significant contributor to death, illness and disability for those in this demographic (AIHW, 2018). Mothers are also at an increased risk of experiencing violence during pregnancy and during the post-partum period (Campo, 2015). Often children are involved in DFV disputes, with 68% of mothers reporting that children had been in their care when they experienced violence from a previous partner (AIHW, 2018).

Women who decide to leave an abusive relationship often believe that they will also be leaving behind the violence (Roberts et al., 2015). However, the post-separation period is one of the most dangerous times for women (Roberts et al., 2015), with women being at an increased risk of being stalked, beaten, harassed or killed by their abusive ex-partner post-separation (Krieger, 2002). In addition, mothers often have little choice but to take part in the family law system to maintain the care of their child(ren) and participate in property and financial settlements (Douglas, 2018b).

## Family court processes

Mothers often engage with the family court system while vulnerable and stressed (Varcoe & Irwin, 2004) to protect themselves and their child(ren) from continued abuse post-separation (DeKeseredy et al., 2004; Gutowski & Goodman, 2020; Varcoe & Irwin, 2004). Part of the role of the family court is to determine residence and contact arrangements (subsequently referred to as custody) of the child(ren). Most English-speaking cultures adopt a legal framework around ‘what’s in the child’s best interest’ (Elizabeth et al., 2012a; Rathus et al., 2019; Rivera et al., 2012a). This is often centred around the protection from harm and abuse to the child, both parents having a meaningful relationship with the child and sharing responsibility for the child (Federal Circuit and Family Court of Australia, 2022b). However, a small body of qualitative research has identified that mothers who have experienced DFV often become distressed by family court processes, in trying to protect themselves and their child(ren) from further harm (Rivera et al., 2012a). This distress has been argued to be caused, in part, from the inadequate and limited training of family court professionals in DFV, the lengthy process of court proceedings, and the distress of mothers having to attend court (Parliament of Australia, 2017).

## Mothers who have experienced DFV and mental health concerns in the family court system

The experience of DFV is associated with the onset and exacerbation of mental health disorders (Salcioglu et al., 2017; Spencer et al., 2019), with a higher risk of developing post-traumatic stress disorder (PTSD; Lagdon et al., 2014; Spencer et al., 2019), depression (Bacchus et al., 2018; Devries et al., 2013; Lagdon et al., 2014), anxiety disorders (Lagdon et al., 2014; Spencer et al., 2019), suicidal ideation and attempted suicide (Devries et al., 2013; Renner & Markward, 2009). Psychological abuse has been found to be a stronger predictor of PTSD symptoms than physical abuse at baseline (Taft et al., 2005). Specifically, denigration appears to be the strongest predictor of PSTD symptoms, suggesting that these behaviours may decrease a woman’s sense of self-worth and wellbeing (Taft et al., 2005). Norwood and Murphy (2012) reported similar findings in relation to denigration, arguing that eroded self-worth may be associated with the recollection of abuse, which therefore inhibits emotional recovery.

Mothers who experience DFV before or during pregnancy are at an increased risk of developing higher levels of post-partum mental health issues (Desmarais et al., 2014). In a perinatal study, Cerulli et al. (2011) found that mothers who reported DFV were more likely to be diagnosed with multiple disorders including, depression, PTSD and panic disorder. Pregnancy and childrearing may potentially increase vulnerability; however, this is not confined to pregnancy and early child rearing (Brown, Conway, et al., 2020). A 10-year longitudinal study of 1507 first-time mothers found that greater depression, anxiety and post-traumatic symptoms, as well as physiological symptoms, including severe headaches, excessive tiredness, back pain and incontinence, were associated with both recent and previous exposure to DFV compared to mothers who had not experienced DFV (Brown, Mensah, et al., 2020).

There is limited research available on the psychological health of mothers who have experienced DFV who are engaged with family court processes. However, research from sexual assault survivors indicates that women who report perceived higher levels of control over family court processes show lower levels of depressive and post-traumatic symptoms (R. M. Walsh & Bruce, 2011). Additionally, Campbell et al. (2001) reported that victims who report hurtful contact with the legal system experience higher psychological and physical distress. Conversely, positive legal outcomes including being heard and acknowledged, and experiencing successful outcomes can lead victim-survivors to feeling a sense of power and validation that can aid recovery and improve their trust in the legal system (Herman, 2003).

Given the prevalence of DFV and the resultant negative psychological outcomes, it is important to examine how family court processes influence mothers’ psychological health during this vulnerable time. Therefore, this scoping review aims to consolidate the existing literature to identify what is known about psychological outcomes for mothers who are engaged in the family court system and who have experienced DFV and what factors contribute to psychological wellbeing and/or distress for these women. In particular, the review sought to answer the following research question, ‘what are the psychological impacts of family court processes on mothers who have experienced DFV?’

## Method

This scoping review evaluated the literature related to mothers with experiences of DFV and their involvement with the family court system. A scoping review aims to provide an initial assessment of the literature, map the available data (Arksey & O’Malley, 2005), identify gaps (Levac et al., 2010) and inform future research, methods or systematic reviews (Pham et al., 2014). Arksey and O’Malley’s (2005) framework was adopted, which recommends using the following steps: identifying the research question, ascertaining relevant articles via an appropriate search strategy, developing the inclusion criteria, charting the data, summarising and reporting the results after collation. A protocol was not registered.

### Search strategy

To identify relevant research for the scoping review, a comprehensive, systematic search of ProQuest and EBSCO were conducted. Additionally, online searches were conducted through the search engines Google Scholar and Griffith University library catalogue. Initial keywords were broad and designed to capture the concepts of DFV, the family court and psychological impact. Backward and forward reference searching of the selected articles was also applied. The search terms employed are outlined in Table 1.

**Table 1.** Database search terms.

Family and domestic violence	Family court	Mother	Psychological outcome
Abuse*	Custody	Women	Psych*
Conflict*	Divorce		Depression
Batter*	Separation		Anxiety
Intimidation*	Judicial		Post-traumatic*
Violence*	Court		Stress
Intimate*	Legal*		Emotional distress
Domestic*	Contact		Psychological*
Coercive*	Residence		Mental*
	Spend*		

Note: Columns were separated by ‘and’, and rows were presented in brackets and separated by ‘or’.

‘*’ was used to define all associated words.

### Inclusion criteria

The following inclusion criteria helped identify articles focusing on the psychological outcomes of mothers who engaged with the family court and had a history of DFV: (a) the article was published in English and peer reviewed, (b) participants were women who had at least one child, (c) participants had experiences of DFV involving the father of their child(ren), (d) participants had attended or were attending custody proceedings, (e) the article reported either original research or secondary analysis of original data, (f) the article was conducted in an English-speaking country, given their similar family legal systems, (g) the article was published during or after the year 2000 to account for changes to the family court, which included the establishment of the Federal Circuit Court in 1999 to help process the workload in a timely manner (Federal Circuit Court and Family Court of Australia, 2022d).

**Figure 1.**
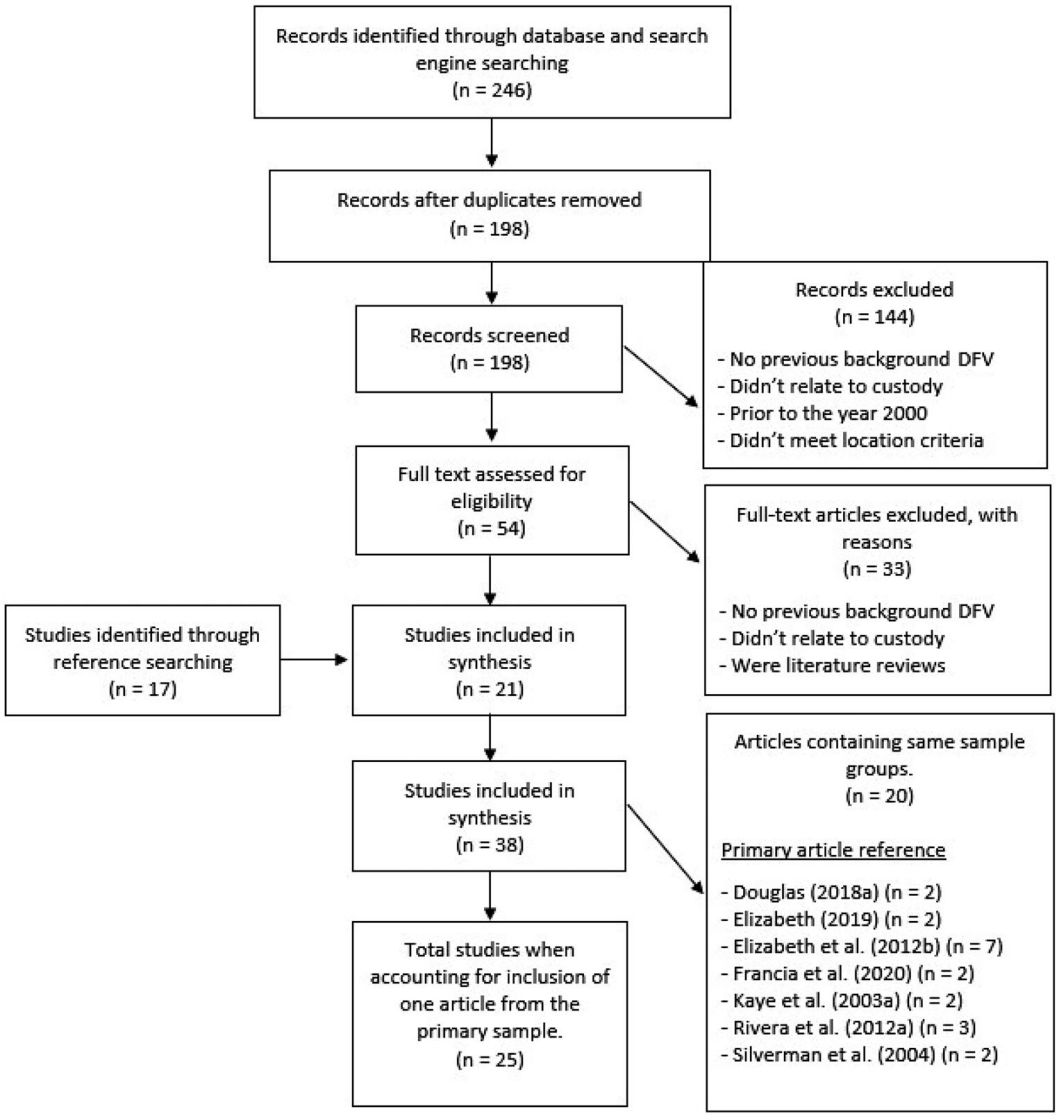
Flowchart of relevant articles identified in search. DFV = domestic and family violence.

The search initially returned 246 articles. Forty-eight duplicate articles were excluded, resulting in a pool of 198 articles. Cursory examination of abstracts and titles resulted in the exclusion of 144 articles as these did not meet the criteria for experiencing DFV, being engaged with family court proceedings, were prior to the year 2000, or did not meet the location criteria. Therefore 54 articles were identified as possibly meeting the inclusion criteria. Two reviewers assessed the remaining articles in depth. Thirty-three articles were excluded as these did not meet the criteria for experiencing DFV, attending custody hearings or were literature reviews. The first author completed citation searching of the identified 21 articles and found a further 17 articles that met the inclusion criteria. Of the 38 articles included in this review, seven primary articles were identified that had subsequent articles reporting findings from the same sample group. Subsequent articles with the same sample were retained if relevant to this review. Therefore, the total number of articles included in this review was 25 (Figure 1).

### Charting data and summarising results

To begin, details relating to location, objective, sample and design of the articles reviewed were extracted by the first author (Table 2). The main themes identified throughout the articles reviewed were completed in five stages as per Braun and Clarke (2006). First, an open approach to coding was undertaken to become familiar with the data by reading and re-reading the articles reviewed, making notes and identifying specific features. Second, the articles reviewed were broken down into segments, paragraphs, lines and key terms before being coded in the database programme Microsoft Excel 2019. Third, emerging themes of the articles reviewed were identified based upon the data and codes. Fourth, these themes were reviewed and checked for consistency from the coded extracts and the dataset. Themes were then named, and a detailed analysis was completed of each. Finally, the themes were examined for relevance to the research question.

**Table 2.** Articles reviewed.

Study number	Author (Year of publication) *Title*	Location	Study objective	Sample *Age (in years)*	Recruitment	Data collection
1	Bemiller (2008) *When battered mothers lose custody: A qualitative study of abuse at home and in the courts.*	USA	Women’s experiences of DFV and institutional violence.	Female (*n* = 16) *27–48*	Flyers, court record searches and snowball sampling	Semi-structured interviews.
2	Coy et al. (2015) *'It’s like going through the abuse again’: Domestic violence and women and children’s (un)safety in private law contact proceedings*	UK	Women’s experiences of DFV and judicial decision making.	Female (*n* = 34) *Age unreported*	Advertisements distributed via women’s specialist organisations and networks	Interviews
3	Douglas (2018a) *Domestic and family violence, mental health and well-being, and legal engagement*	AUS	Women’s experiences of DFV, mental health and legal processes.	Female (*n* = 65) *23–68*	Via DFV support workers or lawyers	Narrative interviews at three time points over 2.5 years.
(3.1)	Douglas (2018b) *Legal systems abuse and coercive control*			As above		
4	Elizabeth (2019) *'It’s an invisible wound’: the disenfranchised grief of post-separation mothers who lose care time*	NZ	Mothers’ involuntary loss of care and shared-care parenting.	Female (*n* = 12) *30s–60s*	Advertisements through women’s centres and networks.	Semi-structured interviews.
(4.1)	Elizabeth (2020) *The affective burden of separated mothers in PA(S) inflected custody law systems: a New Zealand case study*			As above		
5	Elizabeth et al. (2012b) *The gendered dynamics of power in disputes over the postseparation care of children*	NZ	Women’s experiences of DFV and custody proceedings.	Female^a^ (*n* = 21) *20s–50s*	Newspaper advertising and snowball sampling.	Semi-structured interviews.
(5.1)	Elizabeth et al. (2010) *Between a rock and a hard place: Resident mothers and the moral dilemmas they face during custody disputes*			As above		
(5.2)	Elizabeth et al. (2011) *Gendered dynamics in family court counselling*			As above		
(5.3)	Elizabeth et al. (2012a) *‘…He’s just swapped his fists for the system’* *the governance of gender through custody law*			As above		
(5.4)	Tolmie et al. (2010a) *Imposing gender neutral standards on a gendered world: Parenting arrangements in family law post-separation*			As above		
(5.5)	Tolmie et al. (2010b) *Is 50-50 shared card a desirable norm following family separation? Raising questions about current family law practices in New Zealand*			As above		
(5.6)	Tolmie et al. (2009) *Raising questions about the importance of father contact withing current family law practices*			As above		
6	Elizabeth (2015) *From domestic violence to coercive control: Toward the recognition of oppressive intimacy in the family court*	NZ	Women’s experiences of DFV and custody proceedings.	Female (*n* = 1) *Age unreported*		Case study reconstructed from a range of interviews
7	Elizabeth (2017) *Custody stalking: A mechanism of coercively controlling mothers following separation*	NZ	Women’s experiences of post-separation, DFV and custody stalking	Female (*n* = 12) *30s–60s*	Advertisements in women’s local centres and snowball sampling.	Semi-structured interviews.
8	Fitch and Easteal (2018) *Vexatious litigation in family law and coercive control: Ways to improve legal remedies and better protect the victims.*	AUS	Vexatious litigation and its similarities to coercive control.	Female (*n* = 1) *Age unreported*	Participant contacted the researchers and asked to participate.	Unreported
9	Francia et al. (2020) *Mothering* *–* *a mode of protecting rather than parenting in the aftermath of post separation family violence in AUS*	AUS	Women’s experiences of DFV, post separation violence and custody proceedings.	Female (*n* = 36) *34–71*	Invitations via local law firms, national and state organisations, radio interviews, newspaper articles, social networks.	Semi-structured interviews.
(9.1)	Francia et al. (2019) *Addressing family violence post separation* *–* *mothers and fathers’ experiences from AUS*			As above		
10	Gutowski and Goodman (2020) *'Like I'm invisible’: IPV survivor-mothers’ perceptions of seeking child custody through the family court system*	USA	Women’s experiences o DFV and custody proceedings.	Female (*n* = 19) *34–67*	Snowball sampling via professional networks, DV support groups and newsletters.	Structured interviews and legal outcomes questionnaire
11	Hardesty and Ganong (2006) *How women make custody decisions and manage co-parenting with abusive former husbands*	USA	Women’s experiences of DFV, post separation and custody proceedings.	Female (*n* = 19) *21–44*	Identified through court-mandated education programmes for divorcing parents.	Unstructured interviews.
12	Harrison (2008) *Implacable hostile or appropriately protective?*	UK	Women’s experiences of DFV, custody proceeding and post-court abuse	Female (*n* = 70) *16–45*	Recruited from six contact centres	Semi-structured interviews.
13	Kaye et al. (2003a) *Domestic violence and child contact arrangements*	AUS	Women’s experiences of DFV and negotiating custody proceedings.	Female (*n* = 40) *Age unreported*	Recruited via women’s refuges, health centres, and family court.	Semi-structured interviews.
(13.1)	Kaye et al. (2003b) *Domestic violence, separation and parenting: Negotiating safety using legal processes*			As above		
14	Khaw et al. (2021) *'The system had choked me too’: Abused mothers’ perceptions of the custody determination process that resulted in negative custody outcomes*	USA	Women’s experiences of DFV with negative court outcomes.	Female (*n* = 24) *23–48*	Recruited through legal services and supervised visitation programmes.	Secondary analysis of semi-structured interviews.
15	Laing (2017) *Secondary victimization: Domestic violence survivors navigating the family law system*	AUS	Women’s experiences of DFV and the family law system.	Female (*n* = 22) *24–54*	Flyers distributed DV services in Sydney.	Semi-structured interviews.
16	Macdonald (2016) *Domestic violence and private family court proceedings: Promoting child welfare or promoting contact?*	UK	Examines welfare reports of DFV, child welfare and family court.	Families (*n* = 70) *Age not reported*	Reports sampled over 9-month time period from two teams with predetermined criteria.	Document analysis of 70 family welfare reports for the family court.
17	McInnes (2014) *Madness in family law: Mothers’ mental health in the AUS family law system*	AUS	Women’s experience of DFV, mental health and child contact.	(*n* = 4) *Age not reported*	Published family court judgments were generated; of this, four cases were selected.	Published judgements.
18	Miller and Smolter (2011) *'Paper abuse’: When all else fails, batterers use procedural stalking*	USA	Women’s experiences of systems abuse.	Female (*n* = 10) *Age not reported*	Data collected through Women’s Resiliency Project (2009–2011)	Semi-structured interviews.
19	Rathus et al. (2019) *'It’s like standing on a beach, holding your children’s hands, and having a tsunami just coming towards you’: Intimate partner violence and 'expert’ assessments in Australian family law*	AUS	Women’s experiences of DFV and family court reporting.	Female (*n* = 10) *28–46*	Recruited via Women’s Legal Service QLD, IPV organisations, and legal service providers.	Interviews.
20	Rivera et al. (2012a) *Secondary victimisation of abused mothers by family court mediators*	USA	Women’s experiences of DFV and mediation.	Female (*n* = 19) *23–52*	Cases located through dockets that indicated IPV might be present.	Semi-structured interviews and secondary victimisation scale.
(20.1)	Rivera et al. (2012b) *Abused mothers’ safety concerns and court mediators’ custody recommendation*			As above		
(20.2)	Zeoli et al., (2013) *Post-separation abuse of women and their children: Boundary-setting and family court utilization among victimized mothers*			As above		
21	Roberts et al., (2015) *Women’s experiences of the processes associated with the family court of AUS in the context of domestic violence: A thematic analysis*	AUS	Women’s experiences of DFV and the psychological impact of the family court.	Female (*n* = 15) *25–56*	DV service providers, electronic mailing lists and Justice for Children AUS.	Semi-structured interviews and questionnaire.
22	Shepard and Hagemeister (2013) *Perspectives of rural women: Custody and visitation with abusive ex-partners*	USA	Rural women’s experiences, DFV and custody and visitation.	Female (*n* = 23) *20–48*	Advocates of six different domestic violence programmes in towns with populations ranging from 1000 to 14,000 based on 2000 census data.	Focus groups and Use of Children Scale.
23	Silverman et al. (2004) *Child custody determinations in cases involving intimate partner violence: A human rights analysis*	USA	Women’s experiences of DFV and the family court.	Female (*n* = 39) *24–58*	Snowball sampling via social service agencies and legal providers for battered women.	Semi-structured interviews surveys and focus groups.
(23.1)	Slote et al. (2005) *Battered mothers speak out* *–* *Participatory human rights documentation as a model for research and activism in the United States*			As above		
24	Varcoe and Irwin (2004) *'If I killed you, I'd get the kids’: Women’s survival and protection work with child custody and access in the context of women’s abuse*	CAN	Women’s experiences of DFV and custody requirements.	Female (*n* = 27) *21–63*	Word of mouth, flyers, referrals to target women and open house events.	Interviews and focus groups. Documentary evidence provided.
25	Watson and Ancis (2013) *Power and control in the legal system: From marriage/relationship to divorce and custody*	USA	Women’s experiences of DFV and custody proceedings.	Female (*n* = 27) *28 and 59*	Snowball sampling via internet, flyers, personal contacts, a divorce-related listserv/legal website.	Semi-structured interviews.

Note: DFV = domestic and family violence; AUS = Australia; CAN = Canada; NZ = New Zealand; UK = United Kingdom; USA = United States of America; QLD = Queensland; IPV =  Intimate Partner Violence. Articles with multiple papers containing the same sample have been included and bracketed in the ‘study number’ for reference. Only findings relevant to those experiencing DFV were included. Where an article included those who had and had not experienced DFV only findings relevant to those experiencing DFV were included.

^a^Article included participants who had and had not experienced DFV.

### Quality assessment

While Arksey and O’Malley’s (2005) framework does not consist of a quality assessment tool, it has been proposed that in the absence of a critical analysis, scoping reviews may be limited in how the findings are relied upon in evolving policy and practice. Therefore, this review aimed to provide an overview of the quality of the existing research by evaluating the quality of the research as a whole and without excluding any identified articles. This review used the Critical Appraisal Skills Programme, 2022 (CASP) checklists to critically appraise the identified articles (see Supplemental Material).

## Results

Four key themes emerged that summarised how family court processes impacted the psychological health of mothers who had experienced DFV: (a) perpetrators using the system as a mode of coercive control, (b) secondary victimisation as a result of interacting with the system, (c) victims required to relive their abuse, and (d) long-term psychological consequences of having engaged with the system. Of note, most of the articles reviewed were small qualitative studies, based upon the accounts of mothers who experienced DFV and had engaged with the family court, and cannot be understood as literal descriptions of the family court system. Table 3 contains a summary of themes associated with each article reviewed.

**Table 3.** Themes associated with reviewed articles.

Study No.	Theme 1	Theme 2	Theme 3	Theme 4
1	no	yes	no	yes
2	yes	yes	yes	yes
3	yes	no	yes	yes
4	no	yes	no	yes
5	yes	yes	yes	yes
6	yes	yes	no	yes
7	yes	yes	no	yes
8	no	yes	yes	no
9	no	yes	no	yes
10	yes	yes	yes	yes
11	no	yes	no	yes
12	no	yes	no	yes
13	yes	yes	yes	yes
14	no	yes	no	yes
15	yes	yes	yes	yes
16	no	yes	no	no
17	no	yes	no	no
18	yes	yes	no	no
19	no	yes	yes	yes
20	no	yes	yes	yes
21	yes	yes	yes	yes
22	yes	yes	no	yes
23	yes	yes	yes	yes
24	yes	yes	no	yes
25	yes	no	yes	yes

Note*:* Only the primary study reference included. Theme 1: perpetrators using the system as a mode of coercive control; Theme 2: secondary victimisation as a result of interacting with the system; Theme 3: required to relive their abuse; Theme 4: long-term psychological consequences of having engaged with the system.

### Theme 1: perpetrators using the system as a mode of coercive control

The first theme highlights the way that perpetrators engaged with the family court system to further perpetrate abuse. Fourteen articles (2, 3, 5–7, 10, 13, 15, 18, 21–25) reported that fathers engaged in various forms of systems abuse or abuse of process, to intimidate, harass and maintain control over mothers once the relationship had ended. Fathers reportedly prolonged family court cases to financially drain mothers (5, 18, 22–25) and sought increased child custody options to avoid their child support obligations (5, 7, 21, 23–25), to decrease the mothers’ contact with their child(ren) (5, 7, 24, 25) and to reassert their control. These behaviours often went unaddressed by family court professionals (5, 15, 23) with only two articles (2, 13) reporting that the father was classified as a vexatious litigant despite four articles (3, 23–25) reporting the continual nature of bringing orders back to the court unnecessarily. While there appeared to be a lack of awareness of these tactics by family court professionals in the circumstances described in these articles, it should be noted that in Australia vexatious litigation can be difficult to identify in proceedings involving DFV (*Family Law Act 1975* (Cth) Part XIB). In addition, mothers who were experiencing low self-esteem and had experienced trauma were vulnerable to accepting what they perceived to be unfair family court orders (13) perhaps because of a lack of support and ongoing trauma (10).

### Theme 2: secondary victimisation as a result of interacting with the system

Secondary victimisation was a common experience for mothers who were engaged in the family court system. Rivera and colleagues (2012a, p.237) defined secondary victimisation as ‘the negative or unresponsive behaviours by others toward an abuse victim, who experience such response as a further violation of their rights’. Fifteen articles (1, 2, 4–10, 15, 19–21, 23, 24) reported secondary victimisation of mothers by the family court system, which led to increased trauma. Five articles (2, 8, 10, 15, 19) reported that mothers entered the family court system from a place of immense trauma, meaning that they presented in court psychologically ground down (2, 13), depressed (2), stressed (2) and struggling with extreme anxiety (2, 8, 21) following the abuse they had experienced in their previous relationship.

Mothers initially thought that the system would protect them and their children (9, 10, 14), however often found that their experiences of abuse were not validated, which led to feelings of powerlessness and uncertainty (10). Reported abuse claims were minimised (2, 5, 10, 12, 13, 15, 16, 19, 20, 23, 24), dismissed (1, 2, 4, 5, 7, 9, 10, 12, 14–16, 19–21, 23), trivialised (2, 10, 21, 23) and ignored (1, 2, 4, 5, 7, 9, 12, 13, 15, 19, 23, 24) by family court professionals including the mother’s own legal representation (2, 4, 5, 9–11, 15, 21, 22). The behaviours by family court officials extended to accusations of lying when mothers attempted to share their personal experiences of abuse. This meant that mothers were silenced (1, 2, 5, 9, 10, 14, 15, 19, 21), and they experienced feelings of shame (10). This ultimately led to mothers feeling re-victimised by the family court (9, 10, 14, 15, 19, 20). Some mothers felt betrayed by the system that was meant to protect them (10, 14), with one article reporting that PTSD, depression, anxiety and stress were linked to the family court process adding to the trauma of the original abuse (10).

Thirteen articles (1, 2, 5, 9–13, 15, 18, 20–22) reported a lack of knowledge and understanding of DFV by legal professionals. Eight articles (2, 9–11, 15, 20–22) noted that legal professionals were unaware of the complexities involved with coercive control and how these manipulative behaviours were employed by fathers to further control mothers and children (9, 21). This lack of understanding led many legal professionals to blame the victim for the abuse (21, 22).

Ten articles reported that child abuse claims were also minimised or dismissed (1, 2, 4–7, 9, 10, 14, 23). In some instances, mothers were threatened by the perpetrator that they would lose custody if child abuse claims were raised (9), which meant that child abuse allegations often went unaddressed and were not investigated by the family court (4, 9, 10, 23), regardless of documented evidence (1, 10, 23). Some studies reported that in cases where child abuse was present, some mothers were viewed as obstructive (5, 15, 19, 21), vindictive (9, 23), accused of coaching their children into making false allegations (14) or accused of parental alienation (4–6, 9, 15, 19, 23).

Nine articles (2, 5, 9–11, 13, 19, 20, 24) noted the positive experiences that mothers had with the family court. These experiences included feeling heard (9–11, 13, 20), validated (2, 5, 10, 13, 24), safe (20), supported (9, 11, 20), and having their abuse claims correctly documented (19); however, these experiences were rare, and often the children were living full time with the mother or had supervised contact (10). Additionally, when legal professionals had a greater understanding of DFV (2, 9, 24), mothers felt supported (2, 9, 20) and validated (20, 24). Three articles reported that mothers adapted to family court processes and responses after realising that they would need to meet the requirements and expectations of family court officials to protect themselves and their children and therefore worked within the limitations of the system (14, 15, 24).

### Theme 3: required to relive their abuse

This theme highlighted how mothers relived the abuse in family court-mandated processes, which manifested in two different forms: facing the perpetrator in custody negotiations and recounting the abuse to family court professionals.

#### Facing the perpetrator in custody negotiations

Eleven articles (2, 3, 5, 8, 10, 13, 15, 19–21, 23) expressed concerns of mothers being forced to confront their perpetrator in family court and mediation. This requirement extended to having to share a common physical space such as a waiting area (2, 10, 13, 15, 19) with their perpetrator while awaiting proceedings. One article (23) reported that a mother was subjected to face-to-face mediation while a protection order was in place. Mothers reported that their perpetrator often acted aggressively (5, 13, 20), distorted events (25), discounted abuse (2), glared (2) and made inappropriate gestures (13) in family court proceedings.

Upcoming family court appearances caused anxiety (3, 21), fear (21), depression (21), sleep problems (10, 21), lack of concentration (10), tiredness (10) or diarrhoea (10) up to a week before attending court (10). Mothers also reported that being in the same physical space with their perpetrator triggered anxiety (2, 3, 10, 21), panic attacks (8) and intense fear (2, 3, 10, 13, 21, 23). Additionally, leaving the courthouse elicited a high level of fear (2, 10, 13), with two articles reporting that mothers experienced verbal abuse (10, 13) and stalking on the way home (13). Mothers reported that it took days to recover from the confrontation (3), and given the long-term nature of custody proceedings, it meant that mothers struggled to emotionally recover (10) or believed their mental health conditions, including anxiety and depression, were unlikely to improve (21).

There were mixed reactions to safety precautions. Three articles (2, 3, 13) reported that private, secure rooms enhanced feelings of safety; however, two articles (13, 21) reported that mothers were unaware of these secure safety rooms. Two articles (2, 3) reported that staggered arrival times (2) or arriving late (3) helped to minimise facing the perpetrator. However, mothers still had to face their perpetrators inside court. Having a friend, family member or legal representative by their side helped the mother to feel safe (2, 13).

#### Recounting the abuse to family court professionals

Six articles (2, 3, 10, 19–21) reported that mothers were traumatised as a result of providing accounts of abuse in a family court setting. Mothers believed that being in an open court with other people was very traumatic, as it engendered feelings of worthlessness, humiliation, shame and dehumanisation depending upon how family court officials responded (10, 19), especially in the context of sexual abuse in the context of DFV (10).

Three articles (2, 10, 19) reported that mothers were often in the process of making sense of their abusive situation that they had left, and therefore found it difficult to get their story across (3, 10), particularly if they were experiencing PTSD (10). Additionally, mothers often struggled with the preparation of an affidavit due to memory loss (3, 21). One article reported that preparing an affidavit also led to mothers re-experiencing the trauma of the relationship due to the constant recall of abuse (21).

Cross-examination^1^ also emerged as a particularly challenging time. Three articles (2, 3, 8) reported that victims were cross-examined for several hours where they were expected to recount their abusive experiences, which was traumatic. Two articles (3, 8) reported that the father used cross-examination to harass and intimate the mother with no interjection from the judge.

### Theme 4: long-term psychological consequences of having engaged with the system

As family court custody arrangements were finalised, court-mandated changeovers meant that mothers were often forced to come back in contact with their perpetrators to facilitate access to the children of the relationship. Nineteen articles (1, 3–7, 9–15, 19, 20, 22–25) reported that family court-ordered custody arrangements meant that mothers and children were further subjected to post-court abuse.

Five articles (1, 4, 6, 7, 9) reported that mothers experienced high levels of grief associated with lost custody of their children. Feelings of intense grief led to suicidal ideation (9) and attempted suicide (4, 7), as well as extreme emotional pain and diminished mental health (4, 7, 9). High levels of fear (4–6, 9, 11, 15, 22) were also experienced due to the continued abuse directed at mothers post-court (1, 5, 6, 9, 11, 13, 15, 19–21, 23; see Table 4).

**Table 4.** Mothers’ experiences of abuse post-court.

Type of abuse experienced post-court	Article
Physical abuse	1, 5, 11, 13, 15, 19, 20, 21, 23
Emotional abuse	1, 5, 12, 13, 15, 19, 20,
Psychological abuse	1, 5, 6, 13, 19, 20, 25
Verbal abuse	1, 5, 12, 13, 19, 20, 23
Sexual abuse	2, 11, 23
Death threats	1, 13, 20, 23
Being held hostage	13
Threats of father of committing suicide	13
Drugged	13
Stalking	1, 9, 12, 13, 15, 20, 21, 23,
Technological abuse	
Harassing telephone calls	1, 5, 13, 19, 20
Persistent or abusive text messages	5, 6, 9, 20
Persistent or abusive emails	5, 6, 25
Tracking devices placed on the mother	9

In addition, mothers experienced high levels of fear over the safety of their children while in the care of their fathers (9, 11–13, 15, 19, 20, 22–24). Several of the articles reviewed reported accounts of post-court abuse to children as well as the negative psychological impact on children of contact with their father post-court (see Tables 5 and 6).

**Table 5.** Children’s experiences of abuse post-court.

Types of abuse children experienced post-court	Article
Physical abuse	1, 4, 5, 7, 13, 15, 19, 20
Emotional abuse	1, 7, 9, 11, 12, 13, 14, 15, 19, 20, 22 23
Sexual abuse	1, 4, 5, 9, 10, 13, 15, 23
Neglectful parenting	5, 13, 15, 19, 20, 23

**Table 6.** The negative psychological impact of children’s custody with their father post-court.

Psychological impact of custody with their father post-court	Article
Attempted suicide	9, 20
Suicidal ideation	9, 13, 19
Self-injury	5, 20
Post-traumatic stress disorder	9, 19
Depression	9
Anxiety	5, 9, 19, 21
Aggressive behaviour	5, 13,
Naughty behaviour	13, 22
Poor mental health conditions	9, 20
Wetting themselves	1, 5, 13, 22
Unsettled behaviour following contact	4, 5, 13, 15, 22
Nightmares	5, 22
No appetite	13
Sleep problems	13, 22
Stress-related skin reactions	5
Falling academic grades	7, 9, 11
Unable to attend school	9
Low levels of self-confidence	7,
Withdrawn	13
Developmental challenges	9, 22
Struggling with peer relationships	5, 9

While mothers considered their parenting role as protective (4, 5, 9–14, 24), they experienced intense feelings of powerlessness as they not only were unable to protect their children (4, 9, 10, 12, 15, 19, 22, 23), but often had to force their children to attend visitation with their father or risk contravening family court orders (5, 7, 12, 13). Prolonged contact with their perpetrator meant that some mothers were unable to move forward from their experiences of trauma (10).

Five articles (1, 5, 11, 12, 20) reported that mothers adopted survival strategies to help them cope and gain back control over their lives. These included limiting verbal contact with their perpetrator (1, 5, 11, 20) and maintaining physical and psychological distance (5, 11, 13, 20). Seven articles (1, 5, 11, 13, 20, 22, 25) reported that parenting schedules were often used as a form of control, with fathers requiring mothers to be highly flexible in adapting their schedules to fit with their requirements.

Six articles (5, 11, 13, 14, 20, 24) reported that mothers made applications to vary custody arrangements (5, 11, 14) and obtained protection orders to reduce contact (13, 20), to limit abuse. However, three of the articles reviewed reported that mothers lacked the financial and legal resources to bring the orders back to the family court for reassessment (1, 13, 22, 24). Three articles (13, 14, 20) reported that mothers stopped engaging with the system altogether as they felt marginalised and no longer believed in justice.

## Discussion

This scoping review aimed to identify the psychological impact on mothers engaged with the family court system who had experienced DFV. The review of the literature suggested that mothers who engage in the family court system experience a range of short- and long-term psychological impacts and that the court processes facilitate ongoing abuse by the perpetrator. The findings of this review are discussed followed by recommendations that, while proposed for the Australian family law system, may be generalisable to the family law systems of other English-speaking countries.

A key finding of the review was that perpetrators manipulated the legal system to commit further abuse and reassert their control over their previous partner, and this was often not addressed by family court professionals. Motivations for perpetrators prolonging family court cases were to financially impact the mother and to deprive her of contact with her child(ren), which resulted in women feeling overwhelmed and disconnected from their children. Systems abuse has been linked to coercive control and financial abuse (National Domestic and Family Violence Bench Book, 2022). Continual family court applications brought by the perpetrator can place an enormous strain on mothers causing them to ‘give up’ on pursuing orders through the court (Reeves, 2020). While judges often have the power to dismiss all, or part, of a proceeding if they believe that a perpetrator is wrongfully initiating a proceeding, it can be difficult to prove the underlying intent of the perpetrator (Fitch & Easteal, 2018).

Another key finding was that secondary victimisation occurred as a result of engaging in the family court system. Previous research has identified that secondary victimisation has been found to occur in the context of sexual assaults and the resultant criminal proceedings (Herman, 2003). However, this review shows the extent to which it is also an issue for women engaged in the family court system. Secondary victimisation of rape victims has been associated with various symptoms of distress such as post-traumatic stress (Campbell et al., 1999), which is unsurprising as perceived unjust treatment can lead to a negative quality of life (Barkworth & Murphy, 2016). Cattaneo and Goodman (2010) found that empowering experiences in the criminal justice system led to a reduction of depression levels due to victims being able to express their views and be a part of the decision-making process. Events preceding and following victimisation are often incorporated into a victim’s self-narrative of abuse, and therefore the victim requires a fair resolution reflective of the societal views around justice for them to re-establish a sense of control and self-continuity (Pemberton et al., 2019). This review suggests that secondary victimisation in the context of DFV is associated with similar psychological concerns. This review highlighted how secondary victimisation in this context resulted in reduced trust in legal processes and a loss of belief in fairness (Orth, 2002).

Across the articles reviewed, family court professionals were consistently reported to minimise, dismiss, trivialise and ignore claims of abuse by women and their children. Mothers who made claims of child abuse were seen to be obstructive, vindictive and alienating the child from the father. Such claims have been argued to be associated with traditional gendered discourses presenting women as manipulative, hysterical and vindictive with the aim to discredit their claims (Death et al., 2019). Additionally, mothers are often characterised as being mentally ill when there are allegations of coaching or alienating their children, and this can result in mothers being awarded limited contact with their children despite documented abuse (Death et al., 2019).

Our findings also suggested that legal professionals working in the family court system have poor knowledge of DFV and limited understanding of coercive control tactics and how these could be employed by perpetrators. In Australia, research has consistently outlined the need for judicial officers to be regularly trained in DFV and trauma-informed practices with the aim to reduce victim-blaming (Parliament of Australia, 2017). However, these issues seem to extend beyond having a lack of ‘education’ of DFV, with the findings of this review suggesting a judicial bias against women navigating the family court system. As such, improved education of family court officials are likely to have a limited impact on court experiences. Importantly, the negative experiences in the family court were not shared by all, with several articles reviewed finding rare occurrences of women having positive experiences with the courts, suggesting that it is possible for the system to support women and their children appropriately. However, it must be noted that women who have endured more negative experiences of the family court system may be more likely to come forward and share their experiences and therefore may not be reflective of the system.

One of the primary issues with family court proceedings identified in our review was court orders requiring women to have contact with their perpetrators. This led to a range of psychological and physical impacts in the time prior to, during and after the encounter. A small number of the articles reviewed reported positive benefits of safety precautions such as secure rooms, but not all women interviewed knew about these rooms.

A second issue with family court proceedings was that women were often re-traumatised by having to recount their stories in an open court. A smaller subset identified cross-examination as a particularly challenging experience, especially when it was carried out by the perpetrator. Amendments to the Australian *Family Law Act 1975* (Cth) have banned persona cross-examination in circumstances where allegations of DFV have been raised (Attorney-General’s Department, 2021). This ban recognised that survivors were at an increased risk of re-traumatisation through direct cross-examination when perpetrators choose to personally cross-examine the victim in family court proceedings (Attorney-General’s Department, 2021).

Overall, this review suggests that engaging with the family court system exacerbated existing symptoms associated with trauma and/or PTSD for women who had experienced DFV and that the psychological impacts of engaging with the family court were long lasting, with our review suggesting that the outcomes of court proceedings often led to post-court abuse. Mothers suffered intense grief not only in relation to losing contact with their children but also through being unable to protect their children while in the care of their father and reported high levels of fear due to continued contact with their perpetrator and/or being ordered to facilitate the perpetrator’s contact with the children. Salcioglu et al. (2017) reported that fear is one of the strongest predictors of PTSD and depression due to the continuing threat of safety and sense of helplessness it engenders. Therefore, mothers who effectively must maintain contact with their perpetrator through custody arrangements may have an increased likelihood of developing or experiencing exaggerated symptoms of PTSD and depression due to the ongoing fear. Additionally, research has suggested that uncontrollability of a stressor can exacerbate helplessness, anxiety and fear (Salcioglu et al., 2017). Mothers often had limited control to adjust parenting schedules to protect themselves and their children.

### Practical implications

In response to addressing mothers reliving abuse through family court processes, Douglas (2021) proposed the introduction of virtual courts, whereby parties can attend court online through teleconferencing software in the safety of their lawyer’s office or with a support person. Although the courts have generally had these capabilities, they have been rarely used (Douglas, 2021). In Australia, the COVID-19 pandemic and subsequent new reforms in the family court have provided courts with an opportunity for virtual hearings to become more widespread, with conferencing protocols more common (Federal Circuit and Family Court of Australia, 2022a). This may afford mothers experiencing DFV the opportunity to process and rebuild their lives without experiencing the trauma of having to constantly face their perpetrator in court. However, virtual courts are not without risks. T. Walsh (2018) raised concerns in an article that explored proceedings via video link in a group of youth justice clients that identified that vulnerable groups were at an increased risk of being unable to understand proceedings and may feel further isolated. In addition, perpetrators may believe that family court proceedings are less serious if they do not need to physically attend a courthouse (T. Walsh, 2018).

Due to the high levels of mental health concerns that mothers experienced in this review and the reoccurrence of abuse towards mothers and their children post-court, it is suggested, in line with Khaw et al.’s (2021) recommendation, that safety and wellbeing checks are regularly completed. These checks may regularly assess both mother and child’s ongoing psychological health and make sure that mothers and children are physically, sexually and psychologically safe from further abuse, as well as providing referrals to other support services if required. In addition, these services may function as a bridge between the mother and the legal system to provide support if custody orders need to be brought back for reassessment. Furthermore, it is important that perpetrators have access to mental health support services. Shorey et al. (2012) found that mental health problems including depression, PTSD, generalised anxiety disorder, social phobia, panic disorder and alcohol and drug disorders were common among male perpetrators, and as the frequency of mental health problems increase so does perpetration. Therefore, it is just as crucial that men have access to these services as it provides positive long-term outcomes for women and their children.

It has become apparent from the findings of this review that women feel victimised by legal professionals. Currently there is an established independent commission (Legal Services Commission Queensland, 2022) that deals with unsatisfactory professional conduct or misconduct for lawyers and law practice employees. However, training about DFV and trauma-informed lawyering is not required in legal training, and this should be required, at least for those lawyers working in areas of law that often involve DFV such as family law (Wangmann et al., 2023). In addition, the Australian Law Reform Commission released a report in December 2021 that has made recommendations of a Federal Judicial Commission to provide oversight of federal court judges, which this review supports (Australian Law Reform Commission, 2022). Of note, however, is that family court report writers are not subjected to the same review processes, and these negative outcomes can be seen directly in the findings of this review. Family court reports are heavily relied upon by judges and pivotal in cases of DFV as they provide an independent outlook of the issues at hand. It is therefore important that family court writers are required to have specific and consistent trauma-informed training in DFV (Parliament of Australia, 2017).

Given the complexity of family court processes and the different avenues that women can be subjected to re-traumatisation, it is suggested that an independent review body be established. The intention of this body would be to randomly select cases each year for review. This would therefore help to inform policy and law reform, identify risks in the system and help identify areas of training for judicial officials and lawyers working in the family courts.

### Limitations and future directions

One limitation of the review was that there was a paucity of available articles that directly assessed psychological health outcomes caused by engagement in the family court system. While there were many articles based on small qualitative studies that provide insight into the experiences of victim-survivors in the family court system, there was a lack of research using diagnostic measures or longitudinal pre/post designs that established cause and effect. Additionally, research has shown that there are several confounding issues that arise when understanding the nature of DFV. Women’s Health Australia (2018) found that adverse childhood experiences, socioeconomic status, health factors and demographic variables are all associated with an increased risk of DFV. DFV also relates to many negative mental health outcomes as mentioned earlier. Given the complexity of DFV and its associated outcomes, it is difficult to directly understand the role that the family court plays on mental health. On this basis it is therefore recommended that future research take a similar approach to the method employed by Cavalcante Borges Jelinic (2020), who employed qualitative interviews and mental health self-assessment tools over two time points to measure psychological health before and after interactions with the family court. Furthermore, it may be useful to include and explore the risk factors (discussed below) to dissect the intricacy of DFV and therefore begin to understand the impact of court proceedings on mental health.

Another limitation of this study was that this review did not explore the relationship between fathers and their engagement with the family court system. Research has argued that family court processes are a source of stress for men; specifically, Barry and Liddon (2020) found that a reduction in child access was correlated to low mental positivity. Additionally, breakdown of the family relationship has been argued to play a central role in men’s suicide (Shiner et al., 2009). Therefore, it is recommended that future research incorporate men’s experiences of their interactions with the family court to provide further understanding around mental health issues and the family court as a whole.

This study highlights the need for a trauma-informed approach to support families during and after the legal process. Research conducted by Carthy et al. (2023) highlights the significance of examining complex PTSD among survivors of DFV and stresses the need to consider factors such as childhood abuse, negative belief systems, mental health issues, emotional regulation difficulties, traumatic threat responses and interpersonal relationship issues. Additionally, survivors of DFV with childhood adversity may also experience worsening mental health and substance misuse following child removal (Broadhurst & Mason, 2020). This may be particularly important when working with people from cultures with intergenerational trauma. Interestingly, the papers included in our review did not sufficiently explore the impact of the court processes for different cultural groups: a direction for future research. The Power Threat Meaning Framework (PTMF) explores the social, cultural and environmental factors contributing to an individual’s distress, empowering them to make sense of their experiences, and proposing alternative ways of understanding and responding to emotional distress (Johnstone et al., 2018a) and is being used to guide practice in health contexts (Chamberlain et al., 2019). Future research is needed to explore whether employing frameworks such as the PTMF in legal contexts assists those engaged with the system.

Other promising interventions/approaches that could help support mothers who have experienced DFV include triaging and case management programmes. One such initiative, the ‘Lighthouse Project’, aims to identify DFV and safety concerns within the family law system through risk screening when an application or response for parenting orders is filed. Initial risk assessment underpins triaging and referral to specialised teams with training in DFV who evaluate the case for the risk level and help arrange additional support and safety measures and case management (Federal Circuit and Family Court of Australia, 2022c). An alternative approach is undertaken by United Kingdom’s Children and Family Court Advisory and Support Service (CAFCASS) which provides independent services to children and their families involved in family court proceedings. Trained officers (mainly social workers) work with families to assess the family and children’s needs and make recommendations to the family court (CAFCASS, 2017). While the focus is not on DFV, each case is managed individually, taking into account the specific needs of the family and children, including those that arise as a result of DFV, to promote well-being and safety. The Ministry of Justice (2020) conducted a report to evaluate the effectiveness of identifying and responding to DFV allegations and found significant systematic problems in the screening and management of risk that resulted in harmful outcomes. CAFCASS (2022) has since conducted audits and reviewed the report’s recommendations for further consideration and implementation. Future research is needed to provide evidence on the effectiveness of such interventions and to ensure that interventions do not cause further harm.

## Conclusion

The findings of this scoping review consolidate the research of mothers’ experiences of navigating the family court and related psychological outcomes and suggest that perpetrators often used the legal system to reassert their control over and further their abuse towards the mother of their children. Secondary victimisation was experienced by mothers when interacting with judicial officials, and legal professionals had a poor understanding of DFV and the controlling tactics used by perpetrators. Mothers were required to be in close proximity and at times had contact with perpetrators to facilitate court orders, which often led to being re-traumatised. Finally, mothers experienced negative psychological outcomes related to post-court abuse. This review identified practical strategies, including the introduction of virtual courts for DFV hearings, wellbeing and mental health checks for both victim-survivor mothers and perpetrators, consistent trauma-informed/DFV training of lawyers and family court report writers, and the establishment of an independent review body to review cases with the intention of informing policy and law report that will likely improve or protect psychological wellbeing during family court processes as well as highlighting the need for courts to adopt trauma-informed approaches.

## Supplementary Material

Supplemental Material

## Footnotes

1 *Note*. Amendments to the *Australian Family Law Act 1975* (Cth), ss 102NA-102NC, have since banned personal cross-examination in certain circumstances of DFV.

## References

[CIT0001] Arksey, H., & O’Malley, L. (2005). Scoping studies: Towards a methodological framework. *International Journal of Social Research Methodology*, *8*(1), 19–32. 10.1080/1364557032000119616

[CIT0002] Attorney-General’s Department. (2021). *Review of direct cross-examination bank – Family Law Act 1975.* Australian Government. https://www.ag.gov.au/families-and-marriage/consultations/review-direct-cross-examination-ban-family-law-act-1975

[CIT0003] Australian Government. (2019). *Family Law Act* 1975. https://www.legislation.gov.au/Details/C2019C00101

[CIT0004] Australian Institute of Health and Welfare. (2018). *Family, domestic and sexual violence in Australia.* https://www.aihw.gov.au/getmedia/d1a8d479-a39a-48c1-bbe2-4b27c7a321e0/aihw-DFV-02.pdf.aspx?inline=true

[CIT0005] Australian Law Reform Commission. (2022). *Without fear of favour: Judicial impartiality and the law on bias.* https://www.alrc.gov.au/wp-content/uploads/2022/08/ALRC-Judicial-Impartiality-138-Final-Report.pdf

[CIT0006] Bacchus, L. J., Ranganathan, M., Watts, C., & Devries, K. (2018). Recent intimate partner violence against women and health: A systematic review and meta-analysis of cohort studies. *BMJ Open*, *8*(7), e019995. 10.1136/bmjopen-2017-019995 PMC6067339 30056376

[CIT0007] Barkworth, J., & Murphy, K. (2016). System contact and procedural justice policing: Improving quality of life outcomes for victims of crime. *International Review of Victimology*, *22*(2), 105–122. 10.1177/02697580156274044

[CIT0008] Barry, J., & Liddon, L. (2020). Child contact problems and family court issues are related to chronic mental health problems for men following family breakdown. *Psychreg Journal of Psychology*, *3*(4), 57–66. https://papers.ssrn.com/sol3/papers.cfm?abstract_id=3818576

[CIT0009] Bemiller, M. (2008). When battered mothers lose custody: A qualitative study of abuse at home and in the courts. *Journal of Child Custody*, *5*(3–4), 228–255. 10.1080/15379410802583742

[CIT0010] Braun, V., & Clarke, V. (2006). Using thematic analysis in psychology. *Qualitative Research in Psychology*, *3*(2), 77–101. 10.1191/1478088706qp063oa

[CIT0011] Broadhurst, K., & Mason, C. (2020). Child removal as the gateway to further adversity: Birth mother accounts of the immediate and enduring collateral consequences of child removal. *Qualitative Social Work*, *19*(1), 15–37. 10.1177/1473325019893412

[CIT0012] Brown, S. J., Conway, L. J., FitzPatrick, K. M., Hegarty, K., Mensah, F. K., Papadopoullos, S., Woolhouse, H., Giallo, R., & Gartland, D. (2020). Physical and mental health of women exposed to intimate partner violence in the 10 years after having their first child: An Australian prospective cohort study of first-time mothers. *BMJ Open*, *10*(12), e040891. 10.1136/bmjopen-2020-040891 PMC7754634 33371030

[CIT0013] Brown, S. J., Mensah, F., Giallo, R., Woolhouse, H., Hegarty, K., Nicholson, J. M., & Gartland, D. (2020). Intimate partner violence and maternal mental health ten years after a first birth: An Australian prospective cohort study of first-time mothers. *Journal of Affective Disorders*, *262*, 247–257. 10.1016/j.jad.2019.11.015 31732279

[CIT0014] CAFCASS. (2017). *Domestic abuse.* Retrieved April 6, 2023, from https://www.cafcass.gov.uk/grown-ups/parents-and-carers/domestic-abuse/

[CIT0015] CAFCASS. (2022). *Annual report and accounts 2021–2022*. Retrieved from https://assets.publishing.service.gov.uk/government/uploads/system/uploads/attachment_data/file/1125431/cafcass-annual-report-accounts-2021-2022-print.pdf.

[CIT0016] Campbell, R., Sefl, T., Barnes, H. E., Ahrens, C. E., Wasco, S. M., & Zaragoza-Diesfeld, Y. (1999). Community services for rape survivors: Enhancing psychological well-being or increasing trauma? *Journal of Consulting and Clinical Psychology*, *67*(6), 847–858. 10.1037//0022-006x.67.6.847 10596507

[CIT0017] Campbell, R., Wasco, S. M., Ahrens, C. E., Sefl, T., & Barnes, H. E. (2001). Preventing the ‘second rape’: Rape survivors’ experiences with community service providers. *Journal of Interpersonal Violence*, *16*(12), 1239–1259. 10.1177/088626001016012002

[CIT0018] Campo, M. (2015). *Domestic and family violence in pregnancy and early parenthood: Overview and emerging interventions*. Australian Institute of Family Studies. https://aifs.gov.au/sites/default/files/publication-documents/cfca-resource-dv-pregnancy_0.pdf

[CIT0019] Carthy, N., Best, D., Heckels, V., Weber, L., & Eberhardt, J. (2023). Complex posttraumatic stress disorder symptoms among midlife to older female survivors of intimate partner violence. *Psychological Trauma*, *15*(2), 331–339. 10.1037/tra0001238 35201835

[CIT0020] Cattaneo, L. B., & Goodman, L. A. (2010). Through the lens of therapeutic jurisprudence: The relationship between empowerment in the court system and well-being for intimate partner violence victims. *Journal of Interpersonal Violence*, *25*(3), 481–502. 10.1177/0886260509334282 19429713

[CIT0021] Cavalcante Borges Jelinic, A. B. (2020). *Migration and domestic violence: Women’s experiences of proving domestic violence as a requirement for permanent residency in Australia* [Unpublished doctoral thesis, The University of Queensland].

[CIT0022] Cerulli, C., Talbot, N. L., Tang, W., & Chaudron, L. H. (2011). Co-occurring intimate partner violence and mental health diagnoses in perinatal women. *Journal of Women’s Health*, *20*(12), 1797–1803. 10.1089/jwh.2010.2201 PMC3278805 21923282

[CIT0023] Chamberlain, C., Gee, G., Brown, S. J., Atkinson, J., Herrman, H., Gartland, D., Glover, K., Clark, Y., Campbell, S., Mensah, F. K., Atkinson, C., Brennan, S. E., McLachlan, H., Hirvonen, T., Dyall, D., Ralph, N., Hokke, S., & Nicholson, J. (2019). Healing the past by nurturing the future-co-designing perinatal strategies for Aboriginal and Torres Strait Islander parents experiencing complex trauma: Framework and protocol for a community-based participatory action research study. *BMJ Open*, *9*(6), e028397. 10.1136/bmjopen-2018-028397 PMC6575864 31189682

[CIT0024] Coy, M., Scott, E., Tweedale, R., & Perks, K. (2015). It’s like going through the abuse again’: Domestic violence and women and children’s (un)safety in private law contact proceedings. *Journal of Social Welfare and Family Law*, *37*(1), 53–69. 10.1080/09649069.2015.1004863

[CIT0025] Critical Appraisal Skills Programme. (2022). *CASP Qualitative Studies Checklist.* Retrieved from https://casp-uk.net/images/checklist/documents/CASP-Qualitative-Studies-Checklist/CASP-Qualitative-Checklist-2018_fillable_form.pdf

[CIT0026] Death, J., Ferguson, C., & Burgess, K. (2019). Parental alienation, coaching and the best interests of the child: Allegations of child sexual abuse in the Family Court of Australia. *Child Abuse & Neglect*, *94*, 104045. 10.1016/j.chiabu.2019.104045 31212247

[CIT0027] DeKeseredy, W. S., Rogness, M., & Schwartz, M. (2004). Separation/divorce sexual assault: The current state of social scientific knowledge. *Aggression and Violent Behavior*, *9*(6), 675–691. 10.1016/j.avb.2003.08.004

[CIT0028] Department of Families, Fairness and Housing State Government of Victoria, Australia. (2018). *What is family violence?* Victorian State Government. https://services.dffh.vic.gov.au/what-family-violence

[CIT0029] Desmarais, S. L., Pritchard, A., Lowder, E. M., & Janssen, P. A. (2014). Intimate partner abuse before and during pregnancy as risk factors for postpartum mental health problems. *BMC Pregnancy and Childbirth*, *14*(1), 132. 10.1186/1471-2393-14-132 24708777 PMC3991915

[CIT0030] Devries, K. M., Mak, J. Y., Bacchus, L. J., Child, J. C., Falder, G., Petzold, M., Astbury, J., & Watts, C. H. (2013). Intimate partner violence and incident depressive symptoms and suicide attempts: A systematic review of longitudinal studies. *PLoS Medicine*, *10*(5), e1001439. 10.1371/journal.pmed.1001439 23671407 PMC3646718

[CIT0031] Dichter, M. E., Thomas, K. A., Crits-Christoph, P., Ogden, S. N., & Rhodes, K. V. (2018). Coercive control in intimate partner violence: Relationship with women’s experience of violence, use of violence, and danger. *Psychology of Violence*, *8*(5), 596–604. 10.1037/vio0000158 30555730 PMC6291212

[CIT0032] Douglas, H. (2018a). Domestic and family violence, mental health and well-being, and legal engagement. *Psychiatry, Psychology, and Law*, *25*(3), 341–356. 10.1080/13218719.2017.1396865 PMC6818274 31984024

[CIT0033] Douglas, H. (2018b). Legal systems abuse and coercive control. *Criminology & Criminal Justice*, *18*(1), 84–99. 10.1177/1748895817728380

[CIT0034] Douglas, H. (2021). *Women, intimate partner violence, and the law* (1st ed.). Oxford University Press.

[CIT0035] Elizabeth, V. (2015). From domestic violence to coercive control: Towards the recognition of oppressive intimacy in the Family Court. *New Zealand Sociology*, *30*(2), 26–43. http://search.proquest.com.libraryproxy.griffith.edu.au/scholarly-journals/domestic-violence-coercive-control-towards/docview/1703203256/se-2

[CIT0036] Elizabeth, V. (2017). Custody stalking: A mechanism of coercively controlling mothers following separation. *Feminist Legal Studies*, *25*(2), 185–201. 10.1007/s10691-017-9349-9

[CIT0037] Elizabeth, V. (2019). It’s an invisible wound’: The disenfranchised grief of post-separation mothers who lose care time. *Journal of Social Welfare and Family Law*, *41*(1), 34–52. 10.1080/09649069.2019.1554788

[CIT0038] Elizabeth, V. (2020). The affective burden of separated mothers in PA(S) inflected custody law systems: A New Zealand case study. *Journal of Social Welfare and Family Law*, *42*(1), 118–129. 10.1080/09649069.2020.1701943

[CIT0039] Elizabeth, V., Gavey, N., & Tolmie, J. (2010). Between a rock and a hard place: Resident mothers and the moral dilemmas they face during custody disputes. *Feminist Legal Studies*, *18*(3), 253–274. 10.1007/s10691-010-9159-9

[CIT0040] Elizabeth, V., Gavey, N., Tolmie, J. (2012a). “… He’s just swapped his fists for the system” The governance of gender through custody law. *Gender & Society*, *26*(2), 239–260. 10.1177/0891243211434765

[CIT0041] Elizabeth, V., Gavey, N., & Tolmie, J. (2012b). The gendered dynamics of power in disputes over the postseparation care of children. *Violence against Women*, *18*(4), 459–481. 10.1177/1077801212452049 22761171

[CIT0042] Elizabeth, V., Tolmie, J., & Gavey, N. (2011). Gendered dynamics in family court counselling. *New Zealand Journal of Counselling*, *31*(2), 1–20. https://ndhadeliver.natlib.govt.nz/delivery/DeliveryManagerServlet?dps_pid=FL12304804

[CIT0043] Federal Circuit and Family Court of Australia. (2022a). *Attending court: Electronic hearings.* https://www.fcfcoa.gov.au/attending-court/electronic-hearings

[CIT0044] Federal Circuit and Family Court of Australia. (2022b). *Family law: Children: Overview.* https://www.fcfcoa.gov.au/fl/children/overview?web=1&wdLOR=c760E08BF-BB6E-4EA1-97B0-0620B53FCA96

[CIT0045] Federal Circuit and Family Court of Australia. (2022c). *Family law: Lighthouse information sheet for parties – Risk screening.* https://www.fcfcoa.gov.au/sites/default/files/2022-11/LHP_Info_Sheet_Parties_FS_1122V1.pdf

[CIT0046] Federal Circuit and Family Court of Australia. (2022d). *Part 2: Overview of the Court.* https://www.fcfcoa.gov.au/fcc-annual-reports/2020-21/part-2

[CIT0047] Fitch, E., & Easteal, P. (2018). Vexatious litigation in family law and coercive control: Ways to improve legal remedies and better protect the victims. *Family Law Review*, *7*, 103–115.

[CIT0048] Francia, L., Millear, P. M., & Sharman, R. R. (2020). Mothering – A mode of protecting rather than parenting in the aftermath of post separation family violence in Australia. *Children Australia*, *45*(2), 109–116. 10.1017/cha.2020.24

[CIT0049] Francia, L., Millear, P., & Sharman, R. (2019). Addressing family violence post separation – Mothers and fathers’ experiences from Australia. *Journal of Child Custody*, *16*(3), 211–235. 10.1080/15379418.2019.1583151

[CIT0050] Gutowski, E., & Goodman, L. A. (2020). Like I’m Invisible’: IPV survivor-mothers’ perceptions of seeking child custody through the Family Court system. *Journal of Family Violence*, *35*(5), 441–457. 10.1007/s10896-019-00063-1

[CIT0051] Hardesty, J. L., & Ganong, L. H. (2006). How women make custody decisions and manage co-parenting with abusive former husbands. *Journal of Social and Personal Relationships*, *23*(4), 543–563. 10.1177/0265407506065983

[CIT0053] Harrison, C. (2008). Implacably hostile or appropriately protective? *Violence against Women*, *14*(4), 381–405. 10.1177/1077801208314833 18359876

[CIT0054] Herman, J. L. (2003). The mental health of crime victims: Impact of legal intervention. *Journal of Traumatic Stress*, *16*(2), 159–166. 10.1023/A:1022847223135 12699203

[CIT0055] Johnstone, L., Boyle, M., Cromby, J., Dillon, J., Harper, D., Kinderman, P., Longden, E., Pilgrim, D., & Read, J. (2018a). *The Power Threat Meaning Framework: Towards the identification of patterns in emotional distress, unusual experiences and troubled or troubling behavior, as an alternative to functional psychiatric diagnosis*. British Psychological Society. Retrieved from www.bps.org.uk/PTM-Main

[CIT0056] Kaye, M., Stubbs, J., & Tolmie, J. (2003a). Domestic violence and child contact arrangements. *Australian Journal of Family Law*, *17*, 93–133. https://www.researchgate.net/publication/228133931_Domestic_Violence_and_Child_Contact_Arrangements

[CIT0057] Kaye, M., Stubbs, J., & Tolmie, J. (2003b). Domestic violence, separation and parenting: Negotiating safety using legal processes. *Current Issues in Criminal Justice*, *15*(2), 73–94. 10.1080/10345329.2003.12036283

[CIT0058] Khaw, L., Bermea, A. M., Hardesty, J. L., Saunders, D., & Whittaker, A. M. (2021). The system had choked me too”: Abused mothers’ perceptions of the custody determination process that resulted in negative custody outcomes. *Journal of Interpersonal Violence*, *36*(9–10), 4310–4334. 10.1177/0886260518791226 30058441

[CIT0059] Krieger, S. (2002). The dangers of mediation in domestic violence cases. *Cardozo Women’s Law Journal*, *8*, 235–259.

[CIT0060] Lagdon, S., Armour, C., & Stringer, M. (2014). Adult experience of mental health outcomes as a result of intimate partner violence victimisation: A systematic review. *European Journal of Psychotraumatology*, *5*(1), 24794. 10.3402/ejpt.v5.24794 PMC4163751 25279103

[CIT0061] Laing, L. (2017). Secondary victimization: Domestic violence survivors navigating the Family Law system. *Violence against Women*, *23*(11), 1314–1335. 10.1177/1077801216659942 27555598

[CIT0062] Legal Services Commission Queensland. (2022). *Legal Services Commission.* https://www.lsc.qld.gov.au

[CIT0063] Levac, D., Colquhoun, H., & O’Brien, K. K. (2010). Scoping studies: Advancing the methodology. *Implementation Science*, *5*, 69. 10.1186/1748-5908-5-69 20854677 PMC2954944

[CIT0064] Macdonald, G. S. (2016). Domestic violence and private family court proceedings: Promoting child welfare or promoting contact? *Violence against Women*, *22*(7), 832–852. 10.1177/1077801215612600 26567294

[CIT0065] McInnes, E. (2014). Madness in family law: Mothers’ mental health in the Australian family law system. *Psychiatry, Psychology and Law*, *21*(1), 78–91. 10.1080/13218719.2013.774688

[CIT0066] Miller, S. L., & Smolter, N. L. (2011). Paper abuse”: When all else fails, batterers use procedural stalking. *Violence against Women*, *17*(5), 637–650. 10.1177/1077801211407290 21531692

[CIT0067] Ministry of Justice. (2020). *Assessing risk of harm to children and parents in private law children cases: Findings from the second national prevalence survey of aggression and violence in the UK.* Retrieved from https://assets.publishing.service.gov.uk/government/uploads/system/uploads/attachment_data/file/895173/assessing-risk-harm-children-parents-pl-childrens-cases-report_.pdf

[CIT0068] National Domestic and Family Violence Bench Book. (2022). *Systems abuse.* National Domestic and Family Violence Bench Book 2022. https://dfvbenchbook.aija.org.au/understanding-domestic-and-family-violence/systems-abuse/

[CIT0069] Norwood, A., & Murphy, C. (2012). What forms of abuse correlate with PTSD symptoms in partners of men being treated for intimate partner violence? *Psychological Trauma*, *4*(6), 596–604. 10.1037/a0025232

[CIT0070] Orth, U. (2002). Secondary victimization of crime victims by criminal proceedings. *Social Justice Research*, *15*(4), 313–325. 10.1023/A:1021210323461

[CIT0071] Parliament of Australia. (2017). 8. *Strengthening the capacity of family law professionals.* Parliament of Australia. https://www.aph.gov.au/Parliamentary_Business/Committees/House/Social_Policy_and_Legal_Affairs/FVlawreform/Report/section?id=committees/reportrep/024109/25166

[CIT0072] Pemberton, A., Aarten, P. G., & Mulder, E. (2019). Stories as property: Narrative ownership as a key concept in victims’ experiences with criminal justice. *Criminology & Criminal Justice*, *19*(4), 404–420. 10.1177/1748895818778320

[CIT0073] Pham, M. T., Rajić, A., Greig, J. D., Sargeant, J. M., Papadopoulos, A., & McEwen, S. A. (2014). A scoping review of scoping reviews: Advancing the approach and enhancing the consistency. *Research Synthesis Methods*, *5*(4), 371–385. 10.1002/jrsm.1123 26052958 PMC4491356

[CIT0074] Rathus, Z., Jeffries, S., Menih, H., & Field, R. (2019). It’s like standing on a beach, holding your children’s hands, and having a tsunami just coming towards you”: Intimate partner violence and “expert” assessments in Australian Family Law. *Victims & Offenders*, *14*(4), 408–440. 10.1080/15564886.2019.1580646

[CIT0075] Reeves, E. (2020). Family violence, protection orders and systems abuse: Views of legal practitioners. *Current Issues in Criminal Justice*, *32*(1), 91–110. 10.1080/10345329.2019.1665816

[CIT0076] Renner, L. M., & Markward, M. J. (2009). Factors associated with suicidal ideation among women abused in intimate partner relationships. *Smith College Studies in Social Work*, *79*(2), 139–154. 10.1080/00377310902830809

[CIT0077] Rivera, E. A., Sullivan, C. M., & Zeoli, A. M. (2012a). Secondary victimization of abused mothers by Family Court mediators. *Feminist Criminology*, *7*(3), 234–252. 10.1177/1557085111430827 25580100 PMC4287987

[CIT0078] Rivera, E. A., Zeoli, A. M., & Sullivan, C. M. (2012b). Abused mothers’ safety concerns and court mediators’ custody recommendations. *Journal of Family Violence*, *27*(4), 321–332. 10.1007/s10896-012-9426-4 23144531 PMC3491813

[CIT0079] Roberts, D., Chamberlain, P., & Delfabbro, P. (2015). Women’s experiences of the processes associated with the Family Court of Australia in the context of domestic violence: A thematic analysis. *Psychiatry, Psychology and Law*, *22*(4), 599–615. 10.1080/13218719.2014.960132

[CIT0080] Salcioglu, E., Urhan, S., Pirinccioglu, T., & Aydin, S. (2017). Anticipatory fear and helplessness predict PTSD and depression in domestic violence survivors. *Psychological Trauma*, *9*(1), 117–125. 10.1037/tra0000200 27710008

[CIT0081] Shepard, M. F., & Hagemeister, A. K. (2013). Perspectives of rural women: Custody and visitation with abusive ex-partners. *Affilia*, *28*(2), 165–176. 10.1177/0886109913490469

[CIT0082] Shiner, M., Scourfield, J., Fincham, B., & Langer, S. (2009). When things fall apart: Gender and suicide across the life-course. *Social Science & Medicine*, *69*(5), 738–746. 10.1016/j.socscimed.2009.06.014 19608323

[CIT0083] Shorey, R. C., Febres, J., Brasfield, H., & Stuart, G. L. (2012). The prevalence of mental health problems in men arrested for domestic violence. *Journal of Family Violence*, *27*(8), 741–748. 10.1007/s10896-012-9463-z 23284227 PMC3532855

[CIT0084] Silverman, J. G., Mesh, C. M., Cuthbert, C. V., Slote, K., & Bancroft, L. (2004). Child custody determinations in cases involving intimate partner violence: A human rights analysis. *American Journal of Public Health*, *94*(6), 951–957. 10.2105/ajph.94.6.951 15249297 PMC1448371

[CIT0085] Slote, K. Y., Cuthbert, C., Mesh, C. J., Driggers, M. G., Bancroft, L., & Silverman, J. G. (2005). Battered mothers speak out: Participatory human rights documentation as a model for research and activism in the United States. *Violence against Women*, *11*(11), 1367–1395. 10.1177/1077801205280270 16204730

[CIT0086] Spencer, C., Mallory, A. B., Cafferky, B. M., Kimmes, J. G., Beck, A. R., & Stith, S. M. (2019). Mental health factors and intimate partner violence perpetration and victimization. *Psychology of Violence*, *9*(1), 1–17. 10.1037/vio0000156

[CIT0087] Taft, C. T., Murphy, C. M., King, L. A., Dedeyn, J. M., & Musser, P. H. (2005). Posttraumatic stress disorder symptomatology among partners of men in treatment for relationship abuse. *Journal of Abnormal Psychology*, *114*(2), 259–268. 10.1037/0021-843X.114.2.259 15869356

[CIT0088] Tolmie, J., Elizabeth, V., & Gavey, N. (2009). Raising questions about the importance of father contact within current family law practices. *New Zealand Law Review*, *4*, 659–694. https://www.researchgate.net/publication/263419513_Raising_Questions_About_the_Importance_of_Father_Contact_Within_Current_Family_Law_Practices

[CIT0089] Tolmie, J., Elizabeth, V., & Gavey, N. (2010a). Imposing gender neutral standards on a gendered world: Parenting arrangements in family law post-separation. *Canterbury Law Review*, *20*, 117–126. http://www.nzlii.org/nz/journals/CanterLawRw/2010/20.html

[CIT0090] Tolmie, J., Elizabeth, V., & Gavey, N. (2010b). Is 50:50 shared care a desirable norm following family separation? Raising questions about current family law practices in New Zealand. *New Zealand Universities Law Review*, *24*(1), 136–166. https://researchspace.auckland.ac.nz/handle/2292/9560

[CIT0091] Varcoe, C., & Irwin, L. G. (2004). “If I killed you, I’d get the kids”: Women’s survival and protection work with child custody and access in the context of woman abuse. *Qualitative Sociology*, *27*(1), 77–99. 10.1023/B:QUAS.0000015545.82803.90

[CIT0092] Walsh, R. M., & Bruce, S. E. (2011). The relationships between perceived levels of control, psychological distress, and legal system variables in a sample of sexual assault survivors. *Violence against Women*, *17*(5), 603–618. 10.1177/1077801211407427 21502115

[CIT0093] Walsh, T. (2018). Video links in youth justice proceedings: When rights and convenience collide. *Journal of Judicial Administration*, *27*(4), 161–181. http://classic.austlii.edu.au/au/journals/UQLRS/2018/3.html

[CIT0094] Wangmann, J., Bartlett, F., Batagol, B., Booth, T., Douglas, H., Kaye, M., & Seear, K. (2023). What is ‘good’ domestic violence lawyering?: Views from specialist legal services in Australia. *International Journal of Law, Policy and the Family*, *37*(1), ebac034. 10.1093/lawfam/ebac034

[CIT0095] Watson, L. B., & Ancis, J. R. (2013). Power and Control in the Legal System: From marriage/relationship to divorce and custody. *Violence against Women*, *19*(2), 166–186. 10.1177/1077801213478027 23446105

[CIT0096] Women’s Health Australia. (2018). Australian longitudinal study on women’s health. Domestic violence, risk factors and health. https://alswh.org.au/wp-content/uploads/2020/03/2018_DomesticViolence_Report.pdf

[CIT0097] World Health Organization. (2013). *Global and regional estimates of violence against women: prevalence and health effects of intimate partner violence and non-partner sexual violence.* https://apps.who.int/iris/bitstream/handle/10665/85239/9789241564625_eng.pdf;jsessionid=54E7209EB22E1DF88A7C44700321A84D?sequence=1

[CIT0098] Zeoli, A. M., Rivera, E. A., Sullivan, C. M., & Kubiak, S. (2013). Post-separation abuse of women and their children: Boundary-setting and family court utilization among victimized mothers. *Journal of Family Violence*, *28*(6), 547–560. 10.1007/s10896-013-9528-7 23956494 PMC3743119

