# Supplementary material for: The psychological impact on mothers who have experienced domestic violence when navigating the family court system: a scoping review
Source: Psychiatr Psychol Law. 2023 Jul 4;31(4):764–91. doi: 10.1080/13218719.2023.2214927 (PMC11305050; doi:10.1080/13218719.2023.2214927)
Supplement: Supplemental Material [file TPPL_A_2214927_SM6372.docx]

## Supplementary Material 1. Critical Appraisal Skills Programme (CASP) Qualitative Checklist

| Study  Number | Question 1 | Question 2 | Question 3 | Question 4 | Question 5 | Question 6 | Question 7 | Question 8 | Question 9 | Question 10 |
| --- | --- | --- | --- | --- | --- | --- | --- | --- | --- | --- |
| 1 | Yes | Yes | Yes | Yes | Yes | Can’t tell | Can’t tell | Yes | Yes | Valuable |
| 2 | Yes | Yes | Yes | Yes | Yes | Can’t tell | Yes | Yes | Yes | Valuable |
| 3 | Yes | Yes | Yes | Yes | Yes | Yes | Yes | Yes | Yes | Valuable |
| 4 | Yes | Yes | Yes | Yes | Yes | Can’t tell | No | Yes | Yes | Valuable |
| 5 | Yes | Yes | Yes | Yes | Yes | Can’t tell | No | Yes | Yes | Valuable |
| 6 | Yes | Yes | Yes | Yes | Yes | Can’t tell | No | Yes | Yes | Valuable |
| 7 | Yes | Yes | Yes | Can’t tell | Yes | Can’t tell | No | Yes | Yes | Valuable |
| 8 | Yes | Yes | Yes | Can’t tell | Can’t tell | Can’t tell | Can’t tell | Can’t tell | Yes | Valuable |
| 9 | Yes | Yes | Yes | Yes | Yes | Yes | Yes | Yes | Yes | Valuable |
| 10 | Yes | Yes | Yes | Yes | Yes | Yes | Yes | Yes | Yes | Valuable |
| 11 | Yes | Yes | Yes | Yes | Yes | Yes | No | Yes | Yes | Valuable |
| 12 | Yes | Yes | Yes | Yes | Yes | Can’t tell | Yes | Can’t tell | Yes | Valuable |
| 13 | Yes | Yes | Yes | Yes | Yes | Can’t tell | No | No | Yes | Valuable |
| 15 | Yes | Yes | Yes | Yes | Yes | Yes | Can’t tell | Yes | Yes | Valuable |
| 16 | Yes | Yes | Yes | Yes | Yes | Yes | Yes | Yes | Yes | Valuable |
| 17 | Yes | Yes | Yes | Yes | Yes | N/A | No | Yes | Yes | Valuable |
| 18 | Yes | Yes | Yes | Yes | Yes | N/A | Can’t tell | Can’t tell | Yes | Valuable |
| 19 | Yes | Yes | Yes | Yes | Yes | Can’t tell | No | No | Yes | Valuable |
| 20 | Yes | Yes | Yes | Yes | Yes | Can’t tell | Yes | Yes | Yes | Valuable |
| 21 | Yes | Yes | Yes | Yes | Yes | Yes | Yes | Yes | Yes | Valuable |
| 22 | Yes | Yes | Yes | Yes | Yes | Yes | No | Yes | Yes | Valuable |
| 23 | Yes | Can’t tell | Yes | Yes | Yes | Can’t tell | Can’t tell | Can’t tell | Can’t tell | Valuable |
| 24 | Yes | Yes | Yes | Yes | Yes | Yes | Yes | Yes | Yes | Valuable |
| 25 | Yes | Yes | Yes | Yes | Yes | Yes | No | Yes | Yes | Valuable |
|  |  |  |  |  |  |  |  |  |  |  |

*Note.* N/A means not applicable.
